# Blocking endogenous retinoic acid degradation induces oral tooth formation in zebrafish

**DOI:** 10.1073/pnas.2321162121

**Published:** 2024-03-06

**Authors:** William R. Jackman, Lyn S. Miranda Portillo, Carol K. Cox, Alison Ambrosio, Yann Gibert

**Affiliations:** ^a^Biology Department, Bowdoin College, Brunswick, ME 04011; ^b^Department of Cell and Molecular Biology, Cancer Center and Research Institute, University of Mississippi Medical Center, Jackson, MS 39216

**Keywords:** cypriniforms, Cyp26 enzymes, retinoic acid, oral tooth, Dollo's Law

## Abstract

According to Dollo’s Law of irreversibility in evolution, a lost structure is usually considered to be unable to reappear in evolution due to the accumulation over time of mutations in the genes required for its formation. Cypriniform fish are a classic model of evolutionary loss because, while they form fully operational teeth in the ventral posterior pharynx, unlike other teleosts, they do not possess oral teeth. Paleontological data show that Cypriniforms, a clade of teleost fish that includes the zebrafish, lost their oral teeth 50 to 100 Mya. In order to attempt to reverse oral tooth loss in zebrafish, we block the degradation of endogenous levels of retinoic acid (RA) using a specific inhibitor of the Cyp26 RA degrading enzymes. We demonstrate the inhibition of endogenous RA degradation is sufficient to restore oral tooth induction as marked by the re-appearance of expression of early dental mesenchyme and epithelium genes such as *dlx2b* and *sp7* in the oral cavity. Furthermore, we show that these exogenously induced oral tooth germs are able to be at least partly calcified. Taken together, our data show that modifications of signaling pathways can have a significant effect on the reemergence of once-lost structures leading to experimentally induced reversibility of evolutionary tooth loss in cypriniforms.

Teeth are a vertebrate innovation that are believed to have first appeared as tooth-like structures in the posterior pharynx of jawless fish more than 500 Mya (1). Subsequently, teeth formed in the oral cavity coinciding with the evolution of jaws (2). Many current and extinct fish possess both oral and pharyngeal teeth, however, specific regional tooth loss can be observed in certain fish species. A well-known example is the case of oral tooth loss in the cypriniforms, the order of fish in which the zebrafish (*Danio rerio*) belongs. Fossil evidence suggests that the zebrafish lineage originally had oral teeth but lost them approximatively 50 to 100 Mya (3) or even earlier, up to 250 Mya, depending on which dating tools are used (4). It has been speculated that oral tooth loss in zebrafish was related to the loss of *dlx2a* and *dlx2b* expression in the oral epithelium (5), but a region of the *dlx2b* zebrafish promoter is able to drive green fluorescent protein (GFP) expression in the oral teeth of a developing non-cypriniform teleost that naturally possesses both oral and pharyngeal teeth, the Mexican tetra *Astyanax mexicanus* (5). This discovery that the zebrafish *dlx2b* promoter is able to retain information to drive gene expression in oral teeth is particularly exciting as it suggests that oral tooth loss might be reversible in cypriniforms as long as the proper inductive signals are restored, which challenges the law of Irreversibility of Evolution proposed by the French paleontologist Louis Dollo (Dollo’s law) in 1893 (6). We previously reported that zebrafish embryos exposed to exogenous retinoic acid (RA) can form supernumerary pharyngeal teeth in the anterior pharynx (7), but only with high doses of exogenous RA. We therefore asked whether an increase in endogenous RA availability would be sufficient to restore *dlx2b* expression in tooth germs in the oral cavity and allow the formation of oral teeth in zebrafish.

## Results and Discussion

**Fig. 1.**
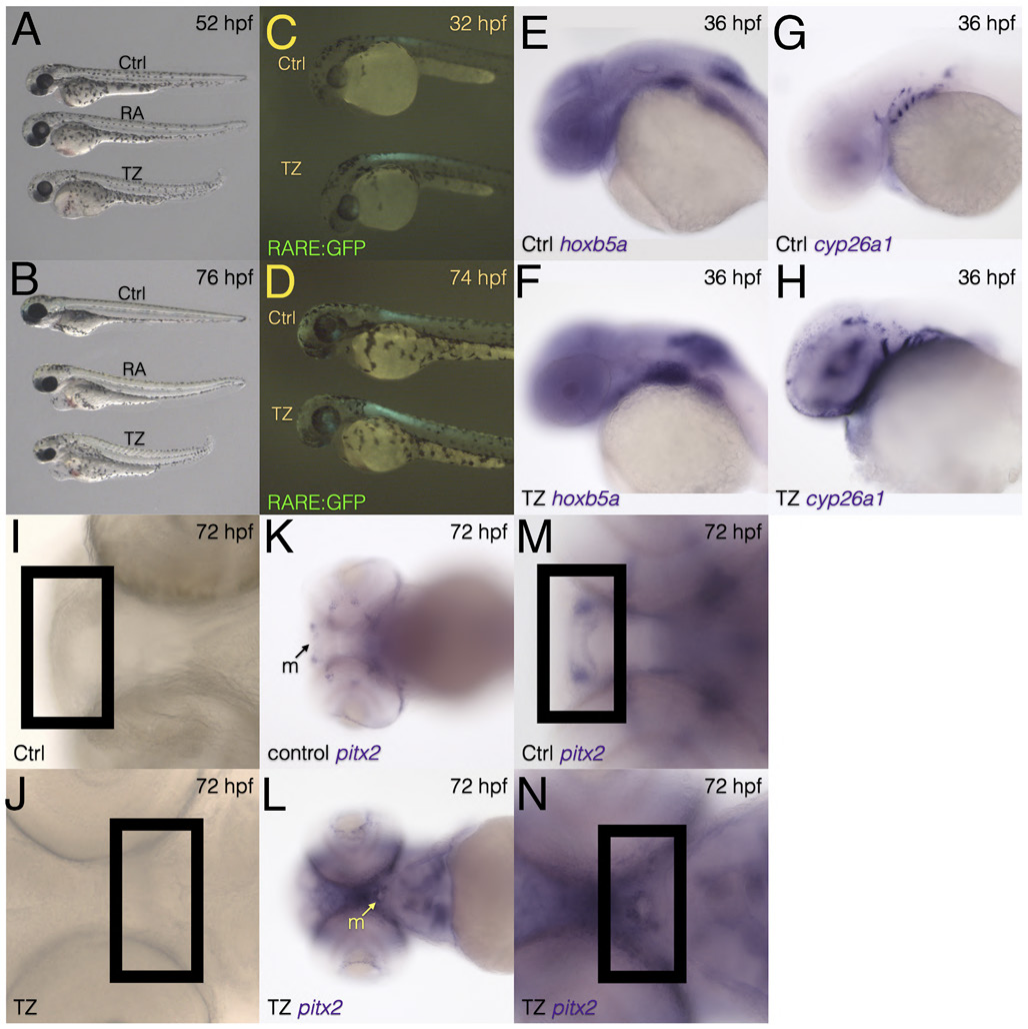
Morphological and gene expression changes induced by TZ exposure. Whole body morphology is similar between embryos exposed to exogenous RA and to TZ starting at 24 hpf (*A* and *B*). Expression levels of a RARE reporter (*C* and *D*), *hoxb5a* (*E* and *F*), and *cyp26a1* (*G* and *H*) are all increase after TZ treatment. Brightfield images (*I* and *J*) and expression of *pitx2* (*K*–*N*) highlight the relatively posterior position of the mouth after TZ is applied. The black box in *I*, *J*, *M*, and *N* denotes the position of the developing mouth.

**Fig. 2.**
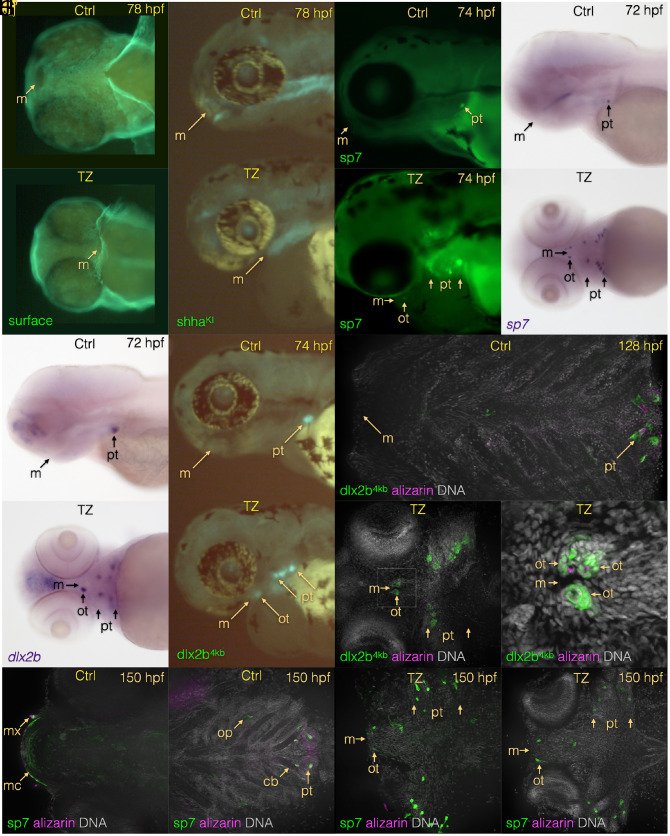
TZ exposure changes the position of the mouth and induces oral tooth formation in zebrafish. TZ treatment starting at 24 hpf results in a posteriorly positioned mouth by 78 hpf (surface staining *A* and *B*; shh^KI^ reporter *C* and *D*). Tooth germs are seen surrounding the mouth as visualized by the *sp7* dental mesenchyme marker (GFP reporter *E* and *F*; mRNA ISH *G* and *H*) and dental epithelium marker *dlx2b* (mRNA ISH *I* and *J*; GFP reporter *K* and *L*). By 128 hpf, compared with a control lacking oral tooth germs (*M*), a TZ-exposed larva exhibits oral dental epithelial reporter expression as well as partial mineralization seen with alizarin red S staining (*N* and *N’*). By 150 hpf, compared with a control lacking oral tooth germs (*O* and *P*), a TZ-exposed larva exhibits oral dental epithelial reporter expression in a more anteriorly positioned mouth (*Q* and *R*). However, at this stage, we detect no evidence of partial mineralization. Boxed region in *N* is zoomed in for *N’*. Abbreviations: cb: ceratobranchial, m = mouth, mc = Meckel’s cartilage, mx = maxilla, op = opercle, ot = oral tooth germs/teeth, pt = pharyngeal tooth germs/teeth.

To test this hypothesis that excess of endogenous RA availability is able to restore oral teeth formation in cypriniforms, we inhibited the activity of the Cyp26 RA degrading enzymes using the selective inhibitor Talarozole [TZ, formerly identified as R115866 (8)] soon after neural crest cell (NCC) ventral migration at 24 hours post fertilization (hpf) (9). TZ has already been successfully used during zebrafish development as a Cyp26 inhibitor (10). We first confirmed that TZ exposure phenocopies what is observed when exogenous RA is applied during zebrafish development in our experimental settings. When exposed to TZ from 24 to 52 hpf and then raised in normal embryonic medium until 76 hpf, zebrafish embryos display all the characteristics that one would expect when embryos are exposed to exogenous RA such as pericardiac edema, small fins, and curved body axis (Fig. 1 *A* and *B*). Such TZ exposure increases GFP expression under the control of RA-responsive elements (RAREs; Fig. 1 *C* and *D*) indicating that RA activity is elevated. At the molecular level, TZ exposure expands anteriorly the expression of the RA-responsive genes *hoxb5a* (Fig. 1 *E* and *F*) and *cyp26a1* (Fig. 1 *G* and *H*). Interestingly, TZ exposure also inhibits or delays the normal migration of the mouth from its original, ventral position as it opens at 60 hpf, to its eventual anterior position achieved by 72 hpf as shown in brightfield images (Fig. 1 *I* and *J*) or as marked by *pitx2a* expression (Fig. 1 *K* and *N*) (11). We confirm the posterior location of the mouth location by surface staining (Fig. 2 *A* and *B*) and using *shha* transgenic GFP knock-in embryos to visualize the mouth opening (Fig. 2 *C* and *D*) and *sp7* reporter GFP transgenic embryos (Fig. 2 *E* and *F*). The presence of tooth germs was examined using the expression of two well-known tooth germ markers expressed in the dental epithelium and mesenchyme, respectively: *sp7* and *dlx2b*. Upon TZ exposure, both genes are expressed adjacent to the posteriorly located mouth (Fig. 2 *G*–*J* arrows) in a pattern consistent with the idea that TZ exposure is able to start the induction of oral tooth germs. Visualization of tooth germs using transgenic embryos expression GFP under the *dlx2b* promoters confirmed that oral tooth germs are initiated under TZ exposure (Fig. 2 *K* and *L*). Because tooth germs were sometimes observed both slightly anterior to and posterior to the ventrally positioned mouth in TZ-exposed embryos (Fig. 2*N'*  ), given the normal morphogenesis of the mouth, we suggest that these may represent both upper and lower jaw tooth germs. However, because jaw elements do not form properly after TZ exposure, similarly to the case with RA overexpression (7), for now, this idea remains a hypothesis. The next question we addressed was the fate of the initiated oral tooth germs under TZ exposure, i.e., do they survive beyond the induction stage and become calcified? To address this question, we double-stained the dlx2b-GFP transgenic embryos exposed to TZ with alizarin red, a dye routinely used to visualize calcified teeth in zebrafish larvae. While we did not detect any calcification during early stages of tooth formation, we were able to detect calcification of oral teeth at 128 hpf, albeit appearing to be at lower than normal levels (Fig. 2 *M* and *N*). Close examination clearly shows the presence of calcification of the tooth placodes in TZ exposed embryos (Fig. 2*N'*  ). At a later stage, 150 hpf, we see no evidence of further tooth calcification. If anything, the development of both oral and pharyngeal tooth germs appears to arrest or even start to degenerate (Fig. 2 *O*–*R*). Interestingly, at 150 hpf in TZ-exposed larvae, the mouth has shifted more anteriorly that at 128 hpf (compare Fig. 2*N* with Fig. 2 *Q* and *R*). Thus, even shifted more toward its normal anterior position, the mouth of a TZ-exposed embryo still possesses oral tooth germs as marked by the *sp7* dental mesenchyme marker (Fig. 2 *Q* and *R*). We conclude that after Cyp26 enzyme inhibition just after the ventral migration of the cranial NCC (after 24 hpf), oral teeth that have been speculated to have been lost for over 50 to 100 My are able to be induced and at least partly calcified in a Cypriniform. This reversal of tooth loss is likely possible because the regulatory regions of tooth development master genes such as *dlx2b* in zebrafish still retain all the genetic information necessary to drive expression in oral teeth in a non-cypriniform fish species (5). Although the loss of individual teeth has been shown to be reversed in mammals, e.g., in lynx after being absent for around 20 My (12), or in birds after having been lost for 70 to 80 My (13), this report demonstrates what could potentially be the oldest experimentally induced tooth loss reversal in animals to date. In a recent paper in mammals, Lynch examined the presence of a second molar (M2) in lynx while all other felids lack M2 (14). Using a developmental model to explain the re-evolution of M2 in lynx, Lynch proposed that developmental programs involved in the formation of serially homologous characters like oral teeth in mammals or pharyngeal and oral teeth in fish may never be lost as long as a single instance of this character still forms and therefore may create what the author qualifies as a loophole in the Dollo’s law (14). As cypriniforms still form teeth, although in a different location than the oral cavity: the pharynx, the genetic information and the “blueprint” to form a tooth exist in Cypriniforms, therefore a re-initiation of the tooth formation cascade by excess RA in the mouth may explain how blocking endogenous RA in Cypriniforms to induce the formation of oral teeth might be seen a violation of Dollo’s law. Our study shows that modifications of a single signaling pathway is sufficient for the re-emergence of lost structure (oral teeth). This re-emergence requires an external intervention which is the suppression of endogenous RA degradation and is therefore not a natural occurring re-emergence of a lost trait. This however did happen in nature in the Guenther’s marsupial frog, where unlike other frog species, teeth can form on the lower jaw, making this example a similar challenger to Dollo’s Law (15). The results of this study, coupled with previous reports in other animal species, strongly challenges Dollo’s Law of Irreversibility of Evolution (6) and illustrates how slightly modifying the action of a single signaling pathway or molecule can be helpful in testing the reversibility of character loss in fish and other animals.

## Materials and Methods

Full Method are presented in *SI Appendix*. Zebrafish embryos were generated using an AB wild-type strain received from the Zebrafish International Resource Center. TZ (R115866) a Cyp26 inhibitor (Sigma SML2092) was dissolved in dimethyl sulfoxide at a stock solution of 10 mM stored at −20 °C or 4 °C.

## Supplementary Material

Appendix 01 (PDF)

## Data Availability

GFP reporter lines used were: dlx2b4kb (Tg(dlx2b:EGFP)), RARE:GFP (Tg(12XRARE-ef1a:gfp), sp7:GFP (Tg(sp7:EGFP)), and shhaKI.shhaKI line was produced by the jackman lab under the name Jackman Lab Genomic feature bo5Tg (http://zfin.org/action/feature/view/ZDB-ALT-230808-17) (16). Detailed descriptions are provided in *SI Appendix*.
