# Supplementary material for: Blocking endogenous retinoic acid degradation induces oral tooth formation in zebrafish
Source: Proc Natl Acad Sci U S A. 2024 Mar 6;121(11):e2321162121. doi: 10.1073/pnas.2321162121 (PMC10945834; doi:10.1073/pnas.2321162121)
Supplement: Supplementary file 1 — Appendix 01 (PDF) [file pnas.2321162121.sapp.pdf]

Supplemental Information:

Zebrafish:

All animal husbandry and experimental techniques were reviewed and approved by the Bowdoin College institutional animal care and use committee and by the UMMC institutional animal care and use committee (IACUC 1161). The zebrafish were handled in accordance with the specified guidelines from both institutional animal care and use committees. Embryos were generated using an AB wildtype strain received from the Zebrafish International Resource Center (ZIRC) and raised in embryonic medium (E3) at 28.5 °C under standard conditions with a 14 h light/10 h dark cycle. GFP reporter lines used were: dlx2b^{4kb} (Tg(dlx2b:EGFP)), RARE:GFP (Tg(12XRARE-ef1a:gfp), sp7:GFP (Tg(sp7:EGFP)), and shha^{KI}.

Chemical exposure:

Retinoic acid (all-trans RA, Sigma R2625) was dissolved in ethanol at stock a concentration of 10^{-4} M and stored at -20°C. Talarozole (TZ or R115866) a Cyp26 inhibitor (Sigma SML2092) was dissolved in DMSO at a stock solution of 10 mM stored at -20°C or 4°C.

Whole Mount *in situ* Hybridization:

Zebrafish embryos exposed to TZ and their respective controls were fixed overnight in 4% paraformaldehyde (PFA) at 4°C. Digoxigenin-labeled insulin riboprobe was transcribed using linearized construct with the T7 RNA polymerase (Roche Diagnostics). Whole-mount *in situ* hybridization (WISH) was performed as described [1].

Histology:

GFP expression in reporter embryos was amplified using an antibody and combined with alizarin red S staining as described in [2]. The 'surface' green staining that highlighted the position of the mouth (Fig. 1A-B) was generated when a batch of embryos was not washed sufficiently to remove surface staining during the antibody labeling procedure.

Microscopy:

WISH stained embryos were photographed using an Axio Imager 2.0 (ZEISS) equipped with an Axiocam 705 color camera (ZEISS). Photographs of head and tooth germ GFP expression in living

zebrafish embryos were taken with a Leica MZ16F stereoscope with a DCF300FX camera. For fluorescence close-ups of the tooth-forming regions, tissues were fixed, and Z-stacks were taken using a Zeiss Axio Imager M2 with an Apotome 2 structured illumination attachment. These stacks were then rendered in 3D and visualized with FluoRender [3]. Colors for fluorescence images were selected to facilitate visibility for diverse vision types [4]. Images were uniformly processed using the Adobe Photoshop software and final figures were assembled using Keynote (Apple Inc.).

Supplemental Information references:

1 Gu, W. *et al.* Using gene-history and expression analyses to assess the involvement of LGI genes in human disorders. *Mol Biol Evol* **22**, 2209-2216 (2005). https://doi.org:10.1093/molbev/msi214

2 Yu, J. C. *et al.* Hedgehog signaling regulates dental papilla formation and tooth size during zebrafish odontogenesis. *Dev Dyn* **244**, 577-590 (2015). https://doi.org:10.1002/dvdy.24258

3 Wan, Y. *et al.* FluoRender: joint freehand segmentation and visualization for many-channel fluorescence data analysis. *BMC Bioinformatics* **18**, 280 (2017). https://doi.org:10.1186/s12859-017-1694-9

4 Wong, B. Points of view: Avoiding color. *Nat Methods* **8**, 525 (2011). https://doi.org:10.1038/nmeth.1642
